# AHR-dependent genes and response to MTX therapy in rheumatoid arthritis patients

**DOI:** 10.1038/s41397-021-00238-4

**Published:** 2021-07-23

**Authors:** Anna Wajda, Ewa Walczuk, Barbara Stypińska, Jakub Lach, Danat Yermakovich, Larysa Sivitskaya, Katarzyna Romanowska-Próchnicka, Tomasz Wysocki, Małgorzata Jarończyk, Agnieszka Paradowska-Gorycka

**Affiliations:** 1 Department of Molecular Biology, National Institute of Geriatrics, Rheumatology and Rehabilitation, Warsaw, Poland; 2 Biobank Lab, Faculty of Biology and Environmental Protection, University of Lodz, Lodz, Poland; 3 BBMRI.pl Consortium, Wrocław, Poland; 4 Institute of Genetics and Cytology, National Academy of Sciences of Belarus, Minsk, Belarus; 5 Department of Systemic Connective Tissue Diseases, National Institute of Geriatrics, Rheumatology and Rehabilitation, Warsaw, Poland; 6 Department of General and Experimental Pathology with Centre for Preclinical Research and Technology (CEPT), Medical University of Warsaw, Warsaw, Poland; 7 Department of Drug Biotechnology and Bioinformatics, National Medicines Institute, Warsaw, Poland

**Keywords:** Genetic counselling, Rheumatic diseases, Gene expression

## Abstract

Methotrexate (MTX) is the first-line therapy for rheumatoid arthritis. Nevertheless, MTX resistance is quite a common issue in clinical practice. There are some premises that aryl hydrocarbon receptor (AhR) gene battery may take part in MTX metabolism. In the present retrospective study, we analyzed genes expression of *AHR* genes battery associated with MTX metabolism in whole blood of RA patients with good and poor response to MTX treatment. Additionally, sequencing, genotyping and bioinformatics analysis of AHR repressor gene (*AHRR*) c.565C > G (rs2292596) and c.1933G > C (rs34453673) have been performed. Theoretically, both changes may have an impact on H3K36me3 and H3K27me3. Evolutionary analysis revealed that rs2292596 may be possibly damaging. Allele G in rs2292596 and DAS28 seems to be associated with a higher risk of poor response to MTX treatment in RA. RA patients with poor response to MTX treatment revealed upregulated *AhR* and *SLC19A1* mRNA level. Treatment with IL-6 inhibitor may be helpful to overcome the low-dose MTX resistance. Analysis of gene expression revealed possible another cause of poor response to MTX treatment which is different from that observed in the case of acute lymphoblastic leukemia.

## Introduction

Methotrexate (MTX) is the most commonly prescribed first-line non-biological, disease-modifying antirheumatic drug and gold standard in RA treatment. Moreover, there are many off-label uses of MTX, such as scleroderma or Crohn’s disease, psoriasis. For oncologic indication, MTX is used in acute lymphocytic and meningeal leukemia, mycosis fungoides, non-Hodgkin lymphomas as well as cancers of the head and neck, breast, and lung, among others. Problems associated with incomplete response or non-response in RA patients are common limitations in the MTX treatment [1]. The reasons for the MTX therapy resistance remain unidentified [2]. Therefore, aryl hydrocarbon receptor (AhR), as a transcription factor of many drug-metabolizing enzymes and drug transporters, may modulate the expression of its target genes and consequently may also lead to a different response to MTX treatment in RA patients. Moreover, MTX is structurally similar to folic acid, which inhibits the overexpression of AHR. It further proves the possible involvement of AHR in MTX metabolism [3]. In 2013, Andrade et al. revealed that in primary MTX resistant cells in acute lymphoblastic leukemia (ALL) AhR is deactivated and its activation by demethylation sensitize cells to MTX and re-express AhR gene [4]. On this point, it is worthy to mention that, methylation is one of the few possible mechanisms of AhR activity regulation. One of them is the auto-regulatory feedback loop. AhR activation induces expression of negative regulators, such as aryl hydrocarbon receptor repressor of AhR (AHRR) which prevent excessive AhR activation. AHRR through the competition with AhR-ARNT complex downregulates expression of AhR-dependent genes [5]. Furthermore, it has been proved that expression of crucial transporters in MTX metabolism such as folate carrier (SLC19A1/RFC1—reduced folate carrier)[6–8] and ABC drug transporters are AhR targets [9–11].

Good response in RA to MTX treatment is followed by few processes, such as induction of apoptosis in T cells or monocyte/macrophage cells, inhibition of the production of proinflammatory cytokines (IL-1 and IL-6), stimulation of the production of anti-inflammatory cytokines (IL-4 and IL-10), and inhibition of metalloproteinases production [12]. Molecular mechanism of MTX metabolism is different in cancer and RA treatment. In cancer, dihydrofolate reductase (DHFR) is competitively inhibited whereas in RA inhibition of DHFR is not the main mechanism but multiple different and complex actions seem to be involved [13, 14]. It has been found that MTX response in RA patients is associated with such genetic variants as 1298A > C (rs1801131) in *MTHFR* [15, 16], 80G > A (rs1051266) in RFC-1 [17], rs2838956A > G and rs7499G > A in *SLC19A1* [18]. Comprehensive analysis of the study on polymorphism associated with MTX response in RA has been described by Qiu et al. [19]. The impact of AhR and its regulatory genes on response to MTX has never been analyzed although the mechanisms of AhR activity regulation may be important. In the present study, both analyzed polymorphisms of *AHRR*, selected on the basis of NGS results, are classified as missense variants. So far c.1933G > C (rs34453673) has only been mentioned in the study on CYP1A2 activity, where the association of this SNP was not proved [20]. In the case of c.565C > G (rs2292596), the association of this variant with RA risk in the Chinese Han population has been analyzed [21] and in few other studies associated with infertility [22, 23] or xenobiotic metabolism [20, 24].

In the case of autoimmune diseases treatment, it is recommended to combine MTX with biologic agents to prolong the duration of their response, probably due to the inhibition of the production of anti-biologic antibodies [25]. Almost 10 years of observations described by Galindo-Rodriguez et al. [26] revealed that after 16 months of DMARD therapy, 50% of treatments had to be discontinued because of inefficacy and/or toxicity and after 4–5 years 75% of therapies had to be discontinued. Generally, the mechanism of drug resistance may be caused by cellular (primary or acquired resistance) or immunological response.

To date, most of the drug transporters and MTX resistance in RA studies were conducted on peripheral blood mononuclear cells (PBMCs) and synovial cells. However, from the clinical point of view, purification of a single immune cell population is usually not feasible and additionally time-consuming. To assess the potential response to MTX treatment the most straightforward approach would be the analysis of whole blood cells. Therefore, in the present project, we characterized the expression profile of selected *AHR* genes battery associated with MTX metabolism in whole blood of RA patients and checked the possibility of predicting response to MTX therapy. Additionally, next-generation sequencing (NGS) of selected genes has been performed. Subsequently, the genetic variants significantly associated with response to MTX therapy have been compared between patients.

## Materials and methods

### Patients

The study was carried out on 158 patients with RA (classified by the American College of Rheumatology/European League Against Rheumatism (EULAR) 2010 criteria, treated with MTX at least 3 months with a dose between 15 and 25 mg per week) treated in the National Institute of Geriatrics, Rheumatology and Rehabilitation in Warsaw, Poland. Blood samples were collected and Larsen score was assessed during hospitalization. Larsen score was assessed by two radiologists. Healthy blood donors (*n* = 94, similar age and gender distribution as RA patients group) as a control group were included in the genotyping study (94 for rs34453673 and 110 for rs2292596 analysis). Fourteen randomly selected patients with good (*n* = 5) and poor (*n* = 9) response to treatment have been included into NGS analysis. Twenty-three patients have been included into gene expression analysis. The research was approved by the Research Ethics Committee of the National Institute of Geriatrics, Rheumatology and Rehabilitation. All the participants included in our study signed informed consent statements. The study was performed in accordance with the 1964 Helsinki declaration and with the ethical standards of our Institute. All subjects were Caucasians.

RA patients were divided into two groups depending on the response to MTX. MTX good response criteria were derived and selected as measures of drug efficacy, they have also been interpreted to represent clinically important improvements according to EULAR “good responder criteria” [27]. Requirements for inclusion in the study were treated with MTX for at least 3 months. The good response drug pattern was carried out for patients with good tolerance of 25 mg per week and no contraindications to MTX. The protocol comprises the following variables: the 28 joint swollen and tender joint number, patient’s global visual analogue scale (VAS), erythrocyte sedimentation rate (ESR), and C reactive protein (CRP) value. The EULAR response criteria are computed using an index of activity in RA, the Disease Activity Scale (DAS28). The DAS28 combines information from the Ritchie Articular Index of 28 joints, the swollen joint number, the ESR, and patient global assessment of their disease activity. The global assessment of disease activity consists of four degrees: remission, low, moderate, high. A 1.2-point change in the DAS28 values from baseline was considered statistically significant. High activity of disease was defined as DAS28 > 5.1, low activity—DAS28 < 3.2, remission—DAS28 < 2.6. Good responders were patients with a significant DAS28 decrease and at least with low disease activity. Good responders (*n* = 9) were treated with MTX or MTX and glucocorticoids. The group of poor responders (*n* = 14) consisted of the patients who suffered from dyspeptic syndromes (*n* = 8) or revealed other symptoms of intolerance such as headache, pancytopenia without efficient results of treatment. Poor responders were treated with tocilizumab or TNFα inhibitors.

### RNA and DNA isolation

Total RNA and DNA was extracted from 500 μl of whole blood using a modified method with microRNA Concentrator kit (A&A Biotechnology, Poland) with Trizol Reagent (Invitrogen, USA) and Blood Mini (A&A Biotechnology, Poland), respectively. The quantity and quality of isolated RNA were evaluated by Quawell Q5000 spectrophotometer. Quantity DNA was calculated by the fluorometric method using HS DNA kit (Invitrogen, USA) and Denovix fluorometer with the final concentration of 4 ng/μl. Quality of the DNA samples was check by the Agilent High Sensitivity DNA reagent kit.

### Next-generation sequencing

Analysis of variants influencing MTX response was conducted using a custom-designed genotyping Ampliseq On-Demand Panel (Illumina, Inc. San Diego, CA, USA) (Table S1). The panel has been designed based on data repositories and the literature. The panel covered all exons and intron/exon boundaries, except the first and fourth exons of the ABCC1 gene with 0.6531 and 0.9468 coverage, respectively. The final concentration of DNA loaded into the flow cell was 9 pM. The NGS was performed on the MiSeq System (Illumina Inc., San Diego, CA, USA). The quality control of raw NGS data was estimated with FASTQC. Aligning sequencing reads was performed in Barrows–Wheeler Aligner against a reference genome NCBIbuild37 (UCSC hg19). The BAM file was annotated by SAMtools v.1.18 with the VCF files generation. Variants were annotated with ANNOVAR using dbSNP IDs, Exome Variant Server, The 1000 Genomes Browser, the Genome Aggregation Database, ClinVar, and REVEL. Variants that were significantly associated with the treatment response in this part of the analysis were further genotyped in the entire patient cohort (section “Genotyping”).

### Bioinformatics analysis

To check the significant SNPs’ impact on the function of associated protein MutationTaster 2.0 has been used (http://www.mutationtaster.org) [28]. Evolutionary analysis of coding SNPs has been conducted using PANTHER-position-specific evolutionary preservation (PSEP) [29]. PANTHER-PSEP calculates the length of time (in millions of years) a given amino acid has been preserved in the lineage leading to the protein of interest. The longer the preservation time, the greater the likelihood of functional impact. In the present analysis we choose the thresholds: “probably damaging” (the preservation time > 450 my), “possibly damaging” (200 my <the preservation time < 450 my) and “probably benign” (the preservation time < 200 my). Additionally, a consensus classifier PredictSNP1.0 (http://loschmidt.chemi.muni.cz/predictsnp1/) was used as the predictor of the SNP effect on protein function that reports results from eight prediction tools (SIFT, PolyPhen-1, PolyPhen-2, MAPP, PhD-SNP, SNAP, PANTHER, ncSNPAnalyzer) as deleterious or neutral along with the percentage predicted accuracy of the result [30] the confidence scores are generated by each tool and the qualitative results (deleterious or neutral), is shown.

### Genotyping

*AHRR* rs34453673 and rs2292596 were analyzed using the TaqMan Genotyping Assays and TaqMan Genotyping Master Mix (Applied Biosystems, USA) on a Quant Studio 5 detection system (Life Technologies, Carlsbad, CA, USA).

### Gene expression

For gene expression analysis pre-validated TaqMan Gene Expression Assays were used: *AhR* (Hs00169233_m1), *ARNT* (Hs01121918_m1), *AHRR* (Hs01005075_m1), *SLC19A1*(Hs00953344_m1), *ABCC1–5* (Hs01561483_m1, Hs00960489_m1, Hs00978452_m1, Hs00988721_m1, Hs00981089_m1), *ABCG2* (Hs01053790_m1), *DHFR* (Hs00758822_s1), *TS* (Hs00426586_m1), and TaqMan Gene Expression Master Mix. Each sample was analyzed in duplicate. Ct value higher than 35 was taken as below quantification. The relative expression was calculated by ΔΔ Ct method or ΔCt method (normalized to GAPDH as reference gene) using Quant Studio 5 real-time PCR System (Applied Biosystems).

### Statistics

Statistical significance between analyzed groups was determined using the non-parametric Mann–Whitney *U* test. A *p* value <0.05 was considered statistically significant. The Fisher test was used to compare differences in allele frequency between compared groups. All calculations were performed using GraphPad Prism 8.4.2.

**Fig. 1:**
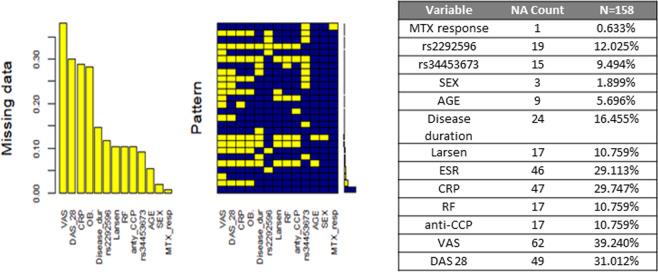
Missing data proportion in multivariable logistic regression model of poor response to MTX treatment probability in RA patients.

Logistic regression model under a codominant, dominant, recessive and overdominant genetic model of inheritance, adjusted by age, in the group of RA patients has been used. Additionally, multivariable logistic regression analysis was conducted to create the best MTX responders model, using the forward and backward variable selection methods (R package MASS). To conduct logistic regression analysis, multiple imputations for missing data was performed using mice R package with default methods. The proportion of missing data in the data set is showed in Fig. 1.

## Results

### Patients

Overall, 158 patients with RA and 94 healthy blood donors were recruited into the study. The clinical and demographic characteristic of RA patients is shown in Table 1. Median age of RA patients was 59 (range 22–89) years. In total, 143 (89.9%) patients were female, the median disease duration was 11 (range 0.5–48) years and the median disease activity score was moderate (DAS28–4.44).

**Table 1:** Characteristic of rheumatoid arthritis patients.

	Median (range)
Age	59 (22–89)
Disease duration (years)	11 (0.5–48)
Female/male	143/16
ESR (mm/h)	24 (0–160)
CRP (mg/l)	11 (0–104)
DAS28	4.44 (0.97–7.51)
VAS (mm)	50 (0–99)
Larsen	3 (0–5)

	*n* (%^a^)
CAD	14 (16)
Anti-CCP positive	116 (81)
RF positive	94 (66)

*ESR* erythrocyte sedimentation rate, *CRP* c-reactive protein, *DAS28* diseases activity score in 28 joints, *VAS* visual analog scale (pain scale), *Larsen* Larsen score of joint involvement on plain radiographs, *CAD* coronary artery disease, *anti-CCP positive* cyclic citrullinated peptide antibodies, *RF positive* rheumatoid factor.

^a^Percentage of available data.

Differences between RA patients with good and poor response to MTX treatment are shown in Table 2. RA poor MTX responders were significantly elder (61 years old) than good responders (56 years old), gender distribution between groups was similar. RA patients with poor MTX response were characterized by significantly higher clinical parameters, such as ESR (*p* < 0.0001), CRP (*p* < 0.0001), and DAS28 (*p* < 0.0001). Among the poor responders, DAS28 was high—5.45, while among patients with a good response to treatment, DAS28 was low—2.65. Additionally, rheumatoid factor was detected more frequently in poor MTX responders than good MTX responders (*p* = 0.002). This difference was not observed in the case of anti-cyclic citrullinated protein antibodies (anti-CCP) occurrence (*p* = 0.32). Moreover, poor responders to MTX were characterized by faster radiological progression. Over 59.6% of poor responders had serious deformations changes in the bones (Larsen over 3) compared to good responders (Larsen score <3 was observed in 57% of patients *p* = 0.049).

**Table 2:** Demographic and clinical characteristic of rheumatoid arthritis patients with poor and good MTX response.

	Poor MTX response	Good MTX response	*p*	
	Median (range)	Median (range)		
Age	61 (25–88)	56 (22–89)	0.008	
Female/male	97 (89%)/11	45 (90%)/5	>0.99^a^	
Disease duration (years)	12 (0–48)	7.5 (0.5–30)	0.028	
ESR (mm/h)	39 (0–160)	15 (1–86)	<0.0001	
CRP (mg/l)	19.50 (1.3–89)	7 (0–104)	<0.0001	
DAS28	5.45 (1.99–7.9)	2.65 (0.97–5.95)	<0.0001	

	*n* (%)	*n* (%)	OR (95% CI)	*p*^a^
Larsen ≥3	69 (60%)	24 (43%)	2 (1.05–3.71)	0.049
RF positive	74	46	3.3 (1.6–7.05)	0.002
Anti-CCP positive	82	75	1.7 (0.7–4.15)	0.32

^a^Fisher test.

### Next-generation sequencing

The distribution of allele frequency of analyzed genes in RA patients with good (*n* = 5) or poor response to MTX treatment (*n* = 9) has been checked (Table 3). Significant differences in allele frequencies of *AHRR* single nucleotide polymorphisms (SNPs) between good and poor responders were revealed. The alternative variant in rs2292596 (*p* = 0.04) and reference variant in rs34453673 (*p* = 0.02) were detected more frequently in poor responders in comparison to good responders.

**Table 3:** Distribution of allele frequency of analyzed genes in rheumatoid arthritis patients with good and poor response to MTX treatment.

Gene	rs number	Nucleotide change	Poor response to MTX treatment		Good response to MTX treatment		*p*^a^
			Reference variant	Alternative variant	Reference variant	Alternative variant	
*MTHFR*	rs1801131	c.1286A > C	12	6	8	2	0.67
*MTHFR*	rs1801133	c.665C > T	16	2	6	4	0.15
*ADORA3*	rs35511654	c.742A > C	15	3	10	0	0.29
*DNMT1*	rs2228612	c.979A > G	14	4	8	2	1
*DNMT1*	rs75616428	c.358G > C	17	1	10	0	1
*DNMT1*	rs61750053	c.206G > A	17	1	10	0	1
*DNMT3A*	–	c.1474 + 2T > G	10	8	6	4	1
*DNMT3A*	–	c.639 + 2 T > G	18	0	10	0	
*AHR*	rs2066853	c.1661G > A	12	6	8	2	0.67
*AHR*	–	c.1662_1663insAG	18	0	10	0	
*AHRR*	–	c.104A > C	18	0	10	0	
*AHRR*	–	c.500A > C	18	0	10	0	
*AHRR*	rs2292596	c.565C > G	8	10	9	1	0.04
*AHRR*	–	c.1418A > C	17	1	8	2	0.53
*AHRR*	–	c.1592A > G	17	1	10	0	1
*AHRR*	rs34453673	c.1933G > C	14	4	3	7	0.02
*ABCC1*	rs45511401	c.2012G > T	17	1	9	1	1
*ABCC2*	–	c.65A > C	18	0	9	1	0.36
*ABCC2*	rs927344	c.116A > T	0	18	0	10	
*ABCC2*	–	c.253C > T	18	0	10	0	
*ABCC2*	–	c.265G > A	18	0	10	0	
*ABCC2*	–	c.706A > T	18	0	10	0	
*ABCC2*	rs2273697	c.1249G > A	12	6	8	2	0.67
*ABCC2*	rs17222561	c.1483A > G	18	0	10	0	
*ABCC2*	rs41318029	c.2761G > A	18	0	10	0	
*ABCC2*	rs17222723	c.3563T > A	17	1	9	1	1
*ABCC2*	rs8187710	c.4544G > A	17	1	9	1	1
*ABCC3*	rs34926034	c.202C > T	18	0	9	1	0.36
*ABCC3*	–	c.614A > C	18	0	10	0	
*ABCC3*	–	c.620A > C	18	0	10	0	
*ABCC3*	rs11568605	c.1037C > T	17	1	10	0	1
*ABCC3*	–	c.2431G > A	18	0	9	1	0.36
*ABCC4*	–	c.3655delA	18	0	10	0	
*ABCC4*	–	c.3458T > G	18	0	10	0	
*ABCC4*	–	c.2992C > T	18	0	10	0	
*ABCC4*	–	c.2560G > T	18	0	9	1	0.36
*ABCC4*	–	c.2468dupA	18	0	10	0	
*ABCC4*	rs3765534	c.2269G > A	17	1	10	0	1
*ABCC4*	–	c.1729T > C	18	0	10	0	
*ABCC5*	–	c.1811T > C	18	0	10	0	
*ABCG1*	–	c.837A > C	17	1	10	0	1
*ABCG2*	rs2231142	c.421C > A	16	2	10	0	0.52
*ABCG2*	rs2231137	c.34G > A	18	0	10	0	
*SLC19A1*	–	c.1322C > T	18	0	10	0	
*SLC19A1*	–	c.949 + 2T > G	18	0	10	0	
*SLC19A1*	–	c.824T > G	18	0	10	0	
*SLC19A1*	rs1051266	c.80A > G	11	7	6	4	1

*MTHFR* methylenetetrahydrofolate reductase, *ADORA3* adenosine A3 receptor, *DNMT1* DNA methyltransferase 1, *DNMT3A* DNA methyltransferase 3 alpha, *AHR* aryl hydrocarbon receptor, *AHRR* aryl hydrocarbon receptor repressor, *ABCC 1–5* ATP-binding cassette subfamily C member 1–5, *ABCG1* ATP-binding cassette subfamily G member 1, *ABCG2* ATP-binding cassette subfamily G member 2, *SLC19A1* solute carrier family 19 member 1.

^a^Fisher exact test two-sided.

### Bioinformatic analysis

Bioinformatic analysis showed that SNPs c.565C > G (rs2292596) and c.1933G > C (rs34453673) in the coding sequence of AHRR protein causes a change in the amino acid sequence, which may affect the protein features. The c.565C > G mutation causes an amino acid substitution from proline to alanine in position 189 of AHRR protein and the c.1933G > C mutation causes substitution in protein sequence from asparagine to histamine in position 645. Both variants rs2292596 and rs34453673 may have an impact on Histone 3 Lysine 36 Tri-Methylation (H3K36me3) and Histone 3 Lysine 27 Tri-Methylation (H3K27me3) regulation. Variant rs34453673 may also regulate RNA Polymerase II (Pol II). In both cases, regions needed for transcriptional repression might be lost.

**Fig. 2:**
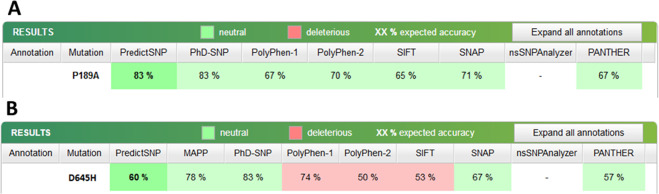
Results of the PredictSNP1 analyzed SNPs in AHRR. **A** rs2292596, **B** rs34453673.

Evolutionary analysis of coding SNPs conducted using PANTHER-PSEP revealed that only rs2292596 in AHRR may be possibly damaging and the preservation time is 324 my while for variant rs34453673 the preservation time is much shorter (30 my). According to the PredictSNP1, it is a neutral polymorphism that may occur in the population (Fig. 2A).

### Distribution of AHRR gene polymorphism in RA and healthy subjects

*AHRR* gene SNPs at position rs34453673 and rs2292596 were analyzed in RA patients and healthy subjects. To analyze the association between *AHRR* gene SNPs and risk of RA, four genetic models, codominant, dominant, recessive, and overdominant were used (Table 4). Genotype frequency in both SNPs was in Hardy–Weinberg equilibrium. We did not reveal an association between analyzed genetic variants in *AHRR* gene and RA risk.

**Table 4:** Distribution of genotypes and allele frequencies of AHRR among patients with rheumatoid arthritis (RA) and healthy subjects.

SNP/genetic model	Genotype/Alleles	RA patients *n* (%)	Healthy subjects *n* (%)	OR (95% CI)	*p* value
*AHRR* rs34453673 (G/C)					
	*Genotype*				
Codominant	GG	58 (36)	36 (38)	Reference	
	GC	74 (46)	47 (50)	1.02 (0.59–1.80)	>0.99
	CC	29 (18)	11 (12)	0.61 (0.29–1.35)	0.32
Dominant	GG	58 (36)	36 (38)	Reference	
	GC + CC	103 (64)	58 (62)	0.91 (0.53–1.52)	0.79
Recesive	CC	29 (18)	11 (12)	1.66 (0.80–3.60)	0.21
	GC + GG	132 (82)	83 (88)	Reference	
Overdominant	GC	74 (46)	47 (50)	0.85 (0.51–1.41)	0.60
	GG + CC	87 (54)	47 (50)	Reference	
	*Alleles*				
	G	190 (59)	119 (63)	Reference	
	C	132 (41)	69 (37)	0.83 (0.58–1.21)	0.35
*AHRR* rs2292596 (C/G)					
Codominant	CC	51 (33)	31 (28)	Reference	
	CG	73 (47)	60 (55)	1.35 (0.77–2.34)	0.32
	GG	31 (20)	19 (17)	1.01 (0.50–2.121)	>0.99
Dominant	CC	51 (33)	31 (28)	Reference	
	CG + GG	104 (67)	79 (72)	1.25 (0.72–2.14)	0.42
Recesive	CC + CG	124 (80)	91 (83)	Reference	
	GG	31 (20)	19 (17)	0.83 (0.45–1.53)	0.63
Overdominant	CG	73 (47)	60 (55)	0.74 (0.46–1.22)	0.26
	CC + GG	82 (53)	50 (45)	Reference	
	Alleles				
	C	175 (56)	122 (55)	Reference	
	G	135 (44)	90 (45)	0.95 (0.67–1.36)	0.86

### Distribution of AHRR gene polymorphism in RA with good and poor response to MTX treatment

To analyze the association between *AHRR* gene SNPs and MTX response in RA patients, four genetic models, codominant, dominant, recessive, and overdominant in the logistic regression model (adjusted by age) were also used (Table 5). Although, in contrast to conducted NGS analysis, we did not reveal a significant association with genotypes, however, we observed that patients with good MTX response revealed more frequently genotype CC in rs34453673 CC genotype (23%) in comparison with the patients with poor MTX response (14%).

**Table 5:** Distribution of genotypes and allele frequencies of AHRR among patients with rheumatoid arthritis RA with good and poor response to MTX treatment adjusted by age.

SNP/genetic model	Genotype	Good responders *n* (%)	Poor responders *n* (%)	OR (95% CI)	*p* value
*AHRR* rs34453673 (G/C)					
Codominant	G/G	15 (32.6)	33 (35.5)	1	0.36
	G/C	20 (43.5)	45 (48.4)	1.0 (0.44–2.32)1	
	C/C	11 (23.9)	15 (16.1)	0.52 (0.18–1.45)	
Dominant	G/G	15 (32.6)	33 (35.5)	1	0.65
	G/CC/C	31 (67.4)	60 (64.5)	0.84 (0.39–1.8)	
Recessive	G/GG/C	35 (76.1)	78 (83.9)	1	0.15
	C/C	11 (23.9)	15 (16.1)	0.51 (0.21–1.27)	
Overdominant	G/GC/C	26 (56.5)	48 (51.6)	1	0.5
	G/C	20 (43.5)	45 (48.4)	1.29 (0.62–2.66)	
	log-Additive	46 (33.1)	93 (66.9)	0.75 (0.45–1.26)	0.27
*AHRR* rs2292596 (C/G)					
Codominant	C/C	17 (37.8)	28 (31.5)	Reference	0.33
	C/G	17 (37.8)	43 (43.8)	1.87 (0.79–4.45)	
	G/G	11 (24.4)	18 (20.2)	1.19 (0.43–3.25)	
Dominant	C/C	17 (37.8)	28 (31.5)	Reference	0.24
	C/GG/G	28 (62.2)	61 (68.5)	1.6 (0.73–3.53)	
Recessive	C/CC/G	34 (75.6)	71 (79.8)	Reference	0.69
	G/G	11 (24.4)	18 (20.2)	0.83 (0.34–2.02)	
Overdominant	C/CG/G	28 (62.2)	46 (51.7)	1	0.15
	C/G	17 (37.8)	43 (48.3)	1.75 (0.81–3.76)	
	log-Additive	45 (33.6)	89 (66.4)	1.15 (0.69–1.92)	0.6

### Association of AHRR rs34453673 and rs2292596 with the clinical phenotype of RA patients

Table 6 summarizes the association of clinical outcome of RA patients with poor or good response to MTX treatment and analyzed *AHRR* genetic variants.

**Table 6:** Association of the AHRR genetic variants and clinical outcome of rheumatoid arthritis patients with poor/good response to MTX treatment.

GENOTYPE rs34453673	Poor MTX response Median (range)	Good MTX response Median (range)	*p*^a^
Disease duration (years)			
CC	15 (0–42)	10.5 (4–23)	0.66
GC	12 (1–39)	9 (1–30)	0.33
GG	13 (0–37)	9 (0.5–20)	0.04
*p*^b^	0.69	0.48	
ESR (mm/h)			
CC	48.56 (9–104)	19.5 (4–86)	0.07
GC	40.5 (3–160)	13 (3–59)	0.005
GG	32 (0–107)	14.50 (0–31)	0.0007
*p*^b^	0.51	CC vs GC: 0.025	
CRP (mg/l)			
CC	24.5 (5–87)	7 (0–104)	0.04
GC	18 (1.3–89)	5 (0–40)	0.003
GG	13.50 (3–75)	9.3 (0–48)	0.06
*p*^b^	0.48	0.65	
DAS28			
CC	5.46 (3.9–7.34)	3.05 (0.97–5.95)	0.005
GC	5.45 (1.99–7.12)	2.65 (1.46–5.89)	<0.0001
GG	5.40 (3.18–7.51)	2.45 (1.5–3.18)	<0.001
*p*^b^	0.92	0.37	
VAS			
CC	73.5 (49–91)	14 (0–82)	0.03
GC	67 (10–87)	15 (3–94)	0.0002
GG	67 (35–99)	24 (6–68)	<0.0001
*p*^b^	0.64	0.71	

	*n* (%)	*n* (%)	*p*^c^
RF positive			
CC	18 (20.2)	7 (15.9)	0.81
GC	40 (44.9)	20 (45.5)	
GG	31 (34.8)	17 (38.6)	
Anti-CCP positive			
CC	23 (21.5)	2 (8)	0.18
GC	48 (44.9)	11 (44)	
GG	36 (33.6)	12 (48)	

GENOTYPE rs2292596	Poor MTX response Median (range)	Good MTX response Median (range)	*p* value
Disease duration (years)			
CC	15 (1–42)	9.5 (1–23)	0.010
GC	12 (1–48)	7 (0.5–30)	0.32
GG	13 (3–37)	7.5 (1–20)	0.055
*p*^b^	0.30	0.61	
ESR (mm/h)			
CC	48.5 (3–160)	15 (1–86))	0.01
GC	32 (0–1144)	19 (5–59)	0.08
GG	35 (8–107)	17.50 (7–36)	0.02
*p*^b^	0.59	0.90	
CRP (mg/l)			
CC	22.5 (3–87)	9 (0–104)	0.12
GC	13 (1.3–89)	8 (1–40)	0.06
GG	12 (3–75)	8.5 (4–15)	0.29
*p*^b^	0.77	0.94	
DAS28			
CC	5.25 (3.11–7.34)	2.85 (0.97–5.95)	<0.0001
GC	5.45 (3.14–7.12)	2.65 (1.88–3.35)	<0.0001
GG	5.48 (4.11–7.51)	2.6 (1.7–3.18)	<0.0001
*p*^b^	0.99	0.41	
VAS			
CC	69 (10–86)	17 (0–82)	0.0009
GC	65 (12–99)	28 (6–52)	<0.0001
GG	67 (50–97)	17 (5–68)	0.004
*p*^b^	0.74	0.92	

	*n* (%)	*n* (%)	*p*^c^
RF positive			
CC	31 (36)	11.2 (26.2)	0.35
GC	39 (45.3)	19 (45.2)	
GG	16 (18.6)	12 (28.6)	
Anti-CCP positive			
CC	36 (35)	7 (28)	0.67
GC	46 (44.7)	11 (44)	
GG	21 (20.4)	7 (28)	

^a^Mann–Whitney test comparison between good and poor MTX responders.

^b^Kruskal–Wallis test with Dunn’s multiple comparison test between different genotypes in analyze group.

^c^Logistic regression in the codominant model.

Our analysis revealed that poor MTX responders, regardless of the analyzed *AHRR* SNPs, were characterized by significantly worse parameters such as ESR, DAS28, and VAS.

Poor MTX responders and carriers of rs34453673 GG genotype were characterized with significantly longer disease duration in comparison to the GG carriers in the good responders’ group (*p* = 0.04). GG carriers revealed the lowest CRP value among patients with poor MTX response (*p* = 0.48). When compared this parameter in poor responders to those observed in the patients with good response to the treatment, the difference was not statistically significant (*p* = 0.06).

Carriers of CC genotype in rs2292596 were characterized by the highest CRP and ESR value in the group of patients with poor response to MTX treatment (*p* = 0.77 and 0.59, respectively). ESR in CC and GG carriers in poor responders was significantly different when compared with good responders (*p* = 0.01 and *p* = 0.02, respectively).

All available clinical and genetic parameters have been included in the model, however, only rs2292596 CG and rs2292596 GG were implemented into the model. At the backward step and at the forward step other genetic variants did not fit the model. Multivariable logistic regression revealed the best model of the probability of poor response to MTX treatment with AIC 80.132 (Table 7) and revealed the significance of rs2292596 CG and DAS28 parameter. Into the model also GG genotype in rs2292596 has been included, however without significance.

**Table 7:** Multivariable logistic regression of probability MTX poor response in rheumatoid arthritis patients model.

	Estimate	Std error	*z* value	pr(>|*z*|)
Intercept	−9.6478	1.8130	−5.321	**1.03e−07**
rs2292596 CG	1.6098	0.7801	2.064	**0.0391**
rs2292596 GG	1.2086	0.9370	1.290	0.1971
DAS28	2.0641	0.3304	6.247	**4.19e−10**
anti-CCP	1.1678	0.7898	1.479	0.1392

### Gene expression

**Fig. 3:**
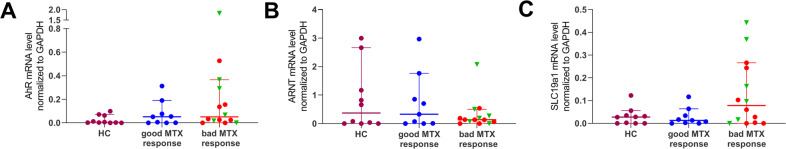
(A) *AhR*, (B) *Arnt*, and (C) *SLC19a1* mRNA level normalized to reference gene (*GAPDH*) in whole blood in the healthy control group (HC, *n* = 10) and RA patients with good response to MTX treatment (*n* = 9) and patients with bad response to MTX treatment (*n* = 14) (red dots—patients with dyspeptic syndromes *n* = 8, green triangles—broader symptoms of MTX intolerance *n* = 6). Results are presented as median with 95% confidential interval. *Significance at *p* < 0.05 (Color figure online).

**Fig. 4:**
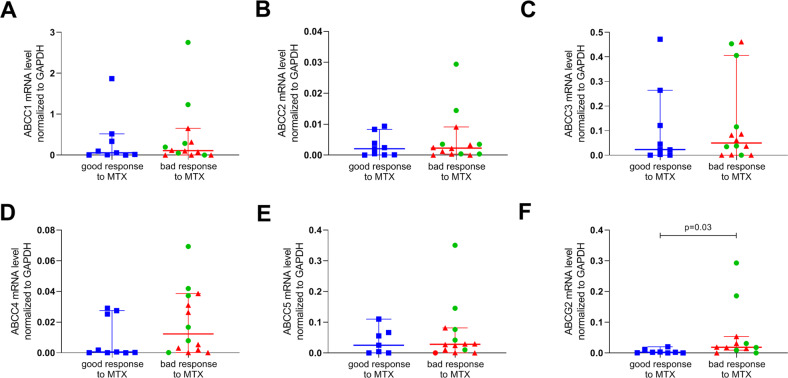
ATP-binding drug transporters mRNA level normalized to reference gene (*GAPDH*) in whole blood in RA patients with good response to MTX treatment (*n* = 9) and patients with poor response to MTX treatment (*n* = 14) (red dots—patients with dyspeptic syndromes *n* = 8, green triangles—broader symptoms of MTX intolerance *n* = 6). Expression of **A** *ABCC1* mRNA level; **B** *ABCC2* mRNA level; **C** *ABCC3* mRNA level; **D** *ABCC4* mRNA level; **E** *ABCC5* mRNA level; **F** *ABCG2* mRNA level. Results are presented as median with 95% confidential interval. *Significance at *p* < 0.05 (Color figure online).

The present study revealed the tendency of higher *AhR* mRNA level in RA patients with poor response to MTX treatment in comparison to patients with good response to MTX treatment (*p* = 0.59) or healthy subjects (*p* = 0.10). We did not reveal statistically significant differences between good MTX responders and healthy subjects (*p* = 0.10) (Fig. 3A). In the case of *ARNT* expression, the reverse trend was observed (Fig. 3B). Healthy subjects and good responders revealed a higher level of *ARNT* expression, without a significant difference between those two groups (*p* = 0.9). Poor responders were characterized by lower mRNA *ARNT* level in comparison to healthy subjects and good responders (*p* = 0.58 and *p* = 0.56, respectively). *AHRR* expression in the group of healthy subjects and RA patients with good response to MTX treatment mostly was below quantification. In RA patients with poor response to MTX treatment, *AHRR* expression was at a low level. Moreover, patients with poor response to MTX treatment were characterized by upregulated *SLC19A1* but not significantly different when compared to healthy subject (*p* = 0.19) or good responders (*p* = 0.20). *SLC19A1* in patients with good response to MTX was at a comparable level as in the healthy subjects (*p* = 0.9) (Fig. 3C). Thymidylate synthase (*TS*) was at below quantification level in the patients with good response to MTX whereas in poor responders expression was detectable. In the case of *DHFR* good responders were characterized by significantly higher mRNA level when compared to poor responders (*p* = 0.007). Additionally, healthy subjects were characterized by low mRNA level of *ABCC1-ABCC5* and *ABCG2* (data not shown). mRNA level of drug transporters *ABCC1, ABCC3*, and *ABCG2* were higher in RA patients with poor response to MTX treatment in comparison to the patients with good response (*p* = 0.69, *p* = 0.81, *p* = 0.74, *p* = 0.08, *p* = 0.69, respectively) (Fig. 4). The difference between groups in *ABCG2* expression was statistically significant (*p* = 0.03) (Fig. 4F). Expression of *ABCC2* and *ABCC5* were at a similar level in both analyzed groups of RA patients.

## Discussion

In the present study, we have revealed significant differences in allele frequency in the case of two polymorphic variants of *AHRR*—rs2292596 and rs34453673 which may have an impact on protein functionality. Further analysis confirmed that only in the case of rs2292596, genetic change might be possibly damaging.

One of the most frequently studied SNPs in *AHRR* is rs2292596 (c.565C > G) and has been reported for the first time by Watanabe et al. [31]. A consequence of this genetic variant occurs just behind the Per–Arnt–Sim region (codon 112–182), which stabilizes the dimerization of AhRR and ARNT complex [24]. Hung et al. concluded that genotype CC is associated with the more active form of AHRR and lower repressor activity over AhR whereas GG—with the less active form of AhRR [24]. Interestingly, more severe forms of endometriosis were found in those with *AhRR* genotype GG (Ala/Ala) in rs2292596 [32] whereas male infertility seems to be associated with wild type CC [33, 22]. Accordingly, with this hypothesis, our NGS analysis has shown that poor MTX responders in RA patients group more frequently carried alternative G allele which supposed to be translated as the less active form of AHRR and higher inducibility of CYP1A2 [34]. Our further analysis included GG (not significant, maybe due to the sample size) and GC variant (statistically significant) in rs2292596 and DAS28 parameter into the model of the probability of poor response to MTX treatment in RA patients. Recently, Cheng et al. evaluated the potential relationship among polymorphisms of *AhR* and aromatic hydrocarbon receptor repressor (*AhRR*), and RA susceptibility in Han Chinese populations. It has been observed that only rs2292596 in *AHRR* is significantly associated with RA risk [21]. Moreover, in the literature, there is no data about this polymorphism and MTX response. In the present study, we did not confirm an association of rs2292596 in *AHRR* with RA risk in Caucasian population. Nevertheless, the main limitation of the current study is a small sample of participants.

According to our NGS analysis, RA patients with poor response to MTX treatment significantly more frequently carried reference allele G in rs34453673 (c.1933G > C) in *AHRR* in comparison with the patients with good response to MTX treatment. In the literature, no data were found on the variant rs34453673 in *AHRR* and its significant association with clinical aspects. In the present study, we did not find an association of rs34453673 in *AHRR* with RA risk in Caucasian population. Our probability model of poor response to MTX treatment in RA patients did not include this genetic variant. However, a consequence of the change in this variant is that polar negatively charged aspartate is replaced by neutral histidine and our computational analysis predicted that this change may affect protein features associated with histone methylation and interaction with polymerase II.

Another aim of the present study was to analyze the expression profile of selected genes associated with the so-called AhR gene battery and MTX response. Does AhR have an impact on a good response to treatment with MTX? We can hypothesize that the answer is yes. First of all, MTX structurally is similar to folic acid which according to the literature interact with AhR [35]. Secondly, in the light of reported studies on MTX resistance or generally on the effect of MTX therapy, it is conceivable that AhR is involved in this drug response [4, 36]. Furthermore, due to AhR’s role in drug metabolism, immune regulations, and RA pathogenesis [37–39], it is interesting to characterize AhR status in RA patients with different response to MTX treatment. Obviously, the present association study because of its methodology cannot find an answer to questions about the mechanisms regulating the proper response to treatment, or if yes why these differences occur, but they can show us interesting new paths for further experimental studies. Typical MTX resistance mechanisms are characterized by decreased activity of folate carrier (SLC19A1/RFC1—reduced folate carrier) and increased level of drug transporters ABCC1–5 and ABCG2. Active transport of MTX into cells depends on SLC19A1 [6] which may be downregulated in an AhR-dependent manner [7]. Also, in PBMCs a positive correlation between *SLC19A1* expression and MTX efficacy has been revealed [8]. The second determinant of unresponsiveness on MTX treatment is the defence role of ATP-binding cassette drug transporters. Another observed mechanism, in cross-resistance to MTX in sulfasalazine resistant T cells, may occur due to inhibition of SLC19A1 (sulfasalazine is its inhibitor) or by induction of ABCG2 expression (MTX is an ABCG2 substrate) [12]. Thus, there are many possible mechanisms of non-response treatment [13, 14].

Surprisingly, in our study, we observed upregulated *AhR* and *SLC19A1* and a significant increase of *ABCG2* mRNA level in the group of patients with poor response to MTX treatment. Other analyzed drug transporters were also at a lower level in comparison with the expression observed in patients with good response to treatment. Active AhR is associated with induction of ABCG2 and drug resistance which is in line with other studies [36, 40–42]. Our analysis of *AHRR* expression revealed very low mRNA level in patients from the group with poor response to MTX and below quantification in RA patients with good response to treatment and also in healthy individuals, regardless of the genotype. Further analysis with the bigger cohort with the protein level analysis and AHR activity is highly recommended. Therefore, we cannot state that poor response (including resistance) to MTX treatment is associated with inactivated AhR as it was described for example in the Andrade et al. study in ALL [4]. In our analyzed group with poor response to MTX treatment, only three patients on 14 were characterized by *AhR* expression at a very low level (Ct value ≥34). Nevertheless, it is also very important to take note that, our group of patients included into gene expression analysis were treated with methylprednisolone and MTX (patients with good response to MTX treatment) or inhibitors of IL-6 or TNFα (poor MTX responders). There are different treatment schemes, and patients selected for gene expression analysis constitute the most homogeneous group in terms of medication. In RA development, IL-6 plays one of the key roles [39] and it has been proved by Hashizume et al. that IL-6 reduced the efficacy of MTX by decreasing the expression of SLC19A1 [43]. Poor responders of MTX in RA group treated with an inhibitor of IL-6 are characterized by a high level of *SLC19A1* and that may be one of the keys on how to overcome MTX resistance? High level of *SLC19A1* in our poor MTX responders may consequently lead to *TS* upregulation and *DHFR* downregulation in comparison with the RA patients with good response to the treatment. Generally, in normal MTX metabolism, polyglutamated MTX inhibits TS. TS is a key protein in dihydrofolate (DHF) synthesis [44]. Therefore, we can assume here, that in our patients with poor response to MTX treatment, high level of *TS*, increased production of DHF which consequently inhibited expression of *DHFR*. One of the limitations of this study is that we did not determine the association of genotypes with gene expression. Furthermore, gene expression dynamically changes over time and depends on many different factors. Limited sample size, time pointing, data access, and financial resources restricted the possibility of multivariate and comprehensive analysis. In the analysis of gene expression, we tried to include the most homogenous group of patients. We tried to focus on the clinical features of poor response and the treatment. Clearly, several important questions remain unanswered and further research should be done to investigate the functional aspect of presented outcomes.

## Conclusions

- Neither rs2292596 nor rs34453673 in *AHRR* is not associated with risk of RA in Caucasian population.
- Allel G in rs2292596 of *AHRR* may increase the probability of poor response in MTX treatment in RA patients although the functional study is necessary.
- Poor response to MTX treatment in RA patients is not associated with inactive AHR.
- Treatment with an inhibitor of IL-6 may have an impact on overcome low-doses MTX resistance.

## Supplementary information

Table 1S
