# Supplementary material for: AHR-dependent genes and response to MTX therapy in rheumatoid arthritis patients
Source: Pharmacogenomics J. 2021 Jul 23;21(5):608–21. doi: 10.1038/s41397-021-00238-4 (PMC8455325; doi:10.1038/s41397-021-00238-4)
Supplement: Supplementary file 1 — Table 1S [file 41397_2021_238_MOESM1_ESM.docx]

Table 1S. Genes included in the custom panel and coverage

| Gene symbol | Protein | Chromosome | Amplicons | Total bases | Percentage coverage | Number exons | Number covered exons |
| --- | --- | --- | --- | --- | --- | --- | --- |
| ABCC1 | ATP-binding cassette, subfamily C, member 1 | chr16 | 44 | 6146 | 99,28% | 31 | 29 |
| ABCC2 | ATP-binding cassette, subfamily C, member 2 | chr10 | 38 | 4958 | 100% | 32 | 32 |
| ABCC3 | ATP-binding cassette, subfamily C, member 3 | chr17 | 44 | 6218 | 100% | 31 | 31 |
| ABCC4 | ATP-binding cassette, subfamily C, member 4 | chr13 | 45 | 5623 | 100% | 32 | 32 |
| ABCC5 | ATP-binding cassette, subfamily C, member 5 | chr3 | 41 | 5850 | 100% | 30 | 30 |
| ABCG1 | ATP-binding cassette, subfamily G, member 1 | chr21 | 29 | 3093 | 100% | 18 | 18 |
| ABCG2 | ATP-binding cassette, subfamily G, member 2 | chr4 | 22 | 2118 | 100% | 15 | 15 |
| ADORA2A | Adenosine A2A receptor | chr22 | 8 | 1339 | 100% | 2 | 2 |
| ADORA3 | Adenosine A3 receptor | chr1 | 7 | 1129 | 100% | 3 | 3 |
| AHR | Aryl hydrocarbon receptor | chr7 | 22 | 2657 | 100% | 11 | 11 |
| AHRR | Arylhydrocarbon receptor repressor | chr5 | 21 | 2760 | 100% | 12 | 12 |
| DHFR | Dihydrofolate reductase | chr5 | 7 | 864 | 100% | 6 | 6 |
| DNMT1 | Dna methyltransferase 1 | chr19 | 50 | 5309 | 100% | 41 | 41 |
| DNMT3A | Dna methyltransferase 3a | chr2 | 29 | 3104 | 100% | 24 | 24 |
| MTHFR | 5,10-methylenetetrahydrofolate reductase | chr1 | 17 | 2081 | 100% | 11 | 11 |
| SLC19A1 | Solute carrier family 19 (folate transporter), member 1 | chr21 | 18 | 2372 | 100% | 7 | 7 |
| TYMS | Thymidylate synthase | chr18 | 10 | 1292 | 100% | 7 | 7 |
